# A Recyclable Palladium-Catalyzed Synthesis of 2-Methylene-2,3-Dihydrobenzofuran-3-ols by Cycloisomerization of 2-(1-Hydroxyprop-2-ynyl)phenols in Ionic Liquids

**DOI:** 10.3390/molecules180910901

**Published:** 2013-09-04

**Authors:** Raffaella Mancuso, Bartolo Gabriele

**Affiliations:** Dipartimento di Chimica e Tecnologie Chimiche, Università della Calabria, Ponte Pietro Bucci 12/C, Arcavacata di Rende (CS) 87036, Italy

**Keywords:** benzofurans, cycloisomerization, dihydrobenzofurans, ionic liquids, palladium, recyclable synthesis

## Abstract

A recyclable palladium-catalyzed synthesis of 2-methylene-2,3-dihydrobenzofuran-3-ols **2** by heterocyclization of 2-(1-hydroxyprop-2-ynyl)phenols **1** in an ionic liquid medium (BmimBF_4_) is presented. The process takes place under relatively mild conditions (100 °C, 5 h) in the presence of catalytic amounts (2 mol %) of PdI_2_ in conjunction with KI (5 equiv with respect to PdI_2_) and an organic base, such as morpholine (1 equiv with respect to **1**), to give **2** in high yields (70%–86%). The PdI_2_-KI catalytic system could be recycled up to six times without appreciable loss of activity. Moreover, products **2** could be easily converted in a one-pot fashion into 2-hydroxymethylbenzofurans **3** (52%–71%, based on **1**) and 2-methoxymethylbenzofurans **4** (52%–80%, based on **1**) by acid-catalyzed allylic isomerization or allylic nucleophilic substitution.

## 1. Introduction

**Scheme 1:**
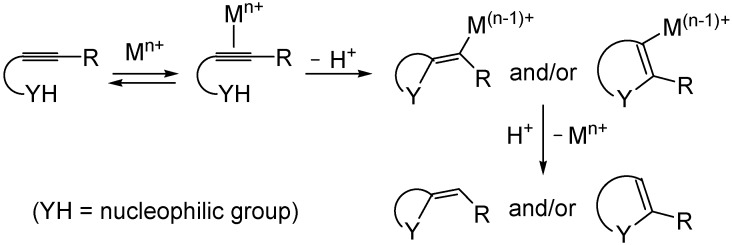
Metal-catalyzed heterocyclization of acetylenes bearing a suitably placed nucleophilic group leading to heterocycles through activation of the triple bond by the metal species followed by intramolecular nucleophilic attack and protonolysis.

Metal-catalyzed heterocyclization reactions are a powerful methodology for the direct synthesis of substituted heterocyclic derivatives starting from readily available substrates [1,2,3,4,5,6,7,8,9,10,11]. The process occurs through the activation of an unsaturated bond by coordination to the metal center, followed by *endo* or *exo* intramolecular attack by the nucleophilic group (YH) and protonolysis or vice versa (Scheme 1—the case of a functionalized alkyne is shown). In particular, we have reported several metal-catalyzed heterocyclizations of acetylenes, bearing a nucleophilic group in a suitable position for cyclization, which have proved valuable for the preparation of a variety of important heterocycles, including furans, pyrroles, thiophenes, and benzothiophenes [12,13,14,15,16,17,18,19,20].

**Scheme 2:**
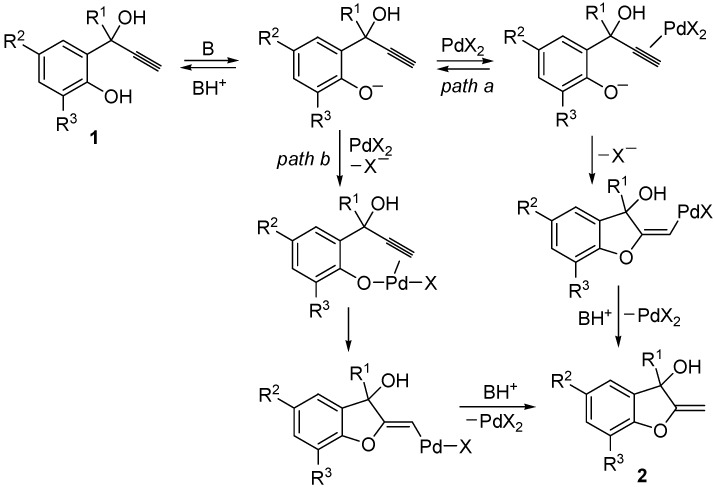
Formation of 2-methylene-2,3-dihydrobenzofuran-3-ols **2** by Pd-catalyzed heterocyclization of 2-(1-hydroxyprop-2-ynyl)phenols **1** in the presence of morpholine as the base (B) in MeOH as the solvent [18].

Recently, we have reported a novel approach to the synthesis of 2-methylene-2,3-dihydrobenzofuran-3-ols **2** by Pd-catalyzed heterocyclization of 2-(1-hydroxyprop-2-ynyl)phenols **1**, carried out in MeOH in the presence of PdX_2_ as catalyst in conjunction with KX (X = Cl, I) and morpholine as the base, necessary for substrate deprotonation [18]. Either 5-*exo*-*dig* intramolecular attack to coordinated triple bond (path *a*) or triple bond insertion into the Pd-O bond of a phenoxypalladium intermediate (path *b*) may take place, according to Scheme 2 [18].

**Scheme 3:**
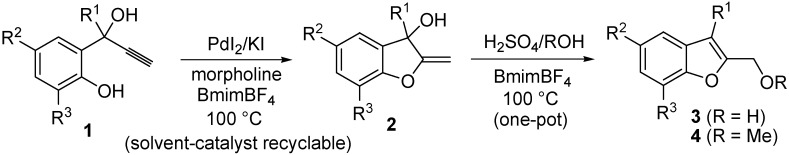
Recyclable Pd-catalyzed synthesis of 2-methylene-2,3-dihydrobenzofuran-3-ols **2** in BmimBF_4_, and their one-pot conversion into 2-hydroxymethylbenzofurans **3** or 2-methoxymethylbenzofurans **4**.

We have now found that this process can also be conveniently performed in an ionic liquid (IL), such as 3-butyl-1-methyl-imidazolium tetrafluoroborate (BmimBF_4_) as the solvent, and that by using this unconventional medium [21,22,23,24,25,26,27,28,29,30,31], it is possible to recycle the catalytic system several times without appreciable loss of activity (Scheme 3). We have also found that, in BmimBF_4_, methylenedihydrobenzofuranols **2** can be readily converted into 2-hydroxymethylbenzofurans **3** or 2-methoxymethylbenzofurans **4** by acid-catalyzed allylic isomerization or allylic nucleophilic substitution in a one-pot fashion. The recyclability of metal catalysts has acquired an increasing importance in the modern organic synthesis, as testified by recent publications in this field [32,33,34,35,36,37,38,39,40,41].

## 2. Results and Discussion

2-(1-Hydroxy-1-phenylprop-2-ynyl)phenol (**1a**) was chosen as the model substrate for testing the reactivity of 2-(1-hydroxyprop-2-ynyl)phenols in ionic liquids. The reaction of **1a**, carried out in BmimBF_4_ as the solvent at 70 °C for 5 h, in the presence of 2 mol % of PdI_2_ in conjunction with KI (5 equiv with respect to PdI_2_) and morpholine (1 equiv. with respect to **1a**), led to the formation of 2-methylene-3-phenyl-2,3-dihydrobenzofuran-3-ol (**2a**) in 60% isolated yield at 80% substrate conversion (Table 1, entry 1). This initial result clearly confirmed the feasibility of the process in an ionic liquid as the reaction medium. Substrate conversion reached 100% either after 8 h at the same temperature (with a **2a** yield of 82%, Table 1, entry 2) or after 5 h at 100 °C (with a **2a** yield of 86%, Table 1, entry 3). The reaction also worked well with 1 mol % of catalyst, as shown in Table 1, entry 4 (**2a** yield was 80%). On the other hand, the use of 4 mol % of KI or the use of PdCl_2_/KCl instead of PdI_2_/KI led to inferior results (Table 1, entries 5 and 6, respectively). Lower yields of **2a** were also obtained working in other ILs, such as BmimCl, BmimPF_6_, BmimN(CN)_2_, or BmimOTf (Table 1, entries 7–10).

The next experiments were aimed at verifying the recyclability of the catalyst-IL system and at generalizing the process to other variously substituted substrates, using BmimBF_4_ as the reaction medium at 100 °C for 5 h. Regarding the recycling experiments, the reaction crude deriving from the reaction carried out under the same conditions as those reported in Table 1, entry 3, was extracted several times with diethyl ether, to isolate the product, while the residue (containing the catalyst dissolved in the IL), after drying under vacuum, was used again by adding to it fresh substrate **1a** and morpholine (1:1 ratio). After stirring at 100 °C for 5 h, **2a** was obtained again, with practically the same yield as the parent reaction (85%, Table 2, entry 1, run 2). The recycling procedure was then repeated up to 5 times, without any appreciable loss of catalytic activity (Table 2, entry 1, runs 3–7).

**Table 1:** Iodocyclodehydration of 4-mercapto-3-methyl-1-phenylpent-1-yn-3-ol (**1a**) to 3-iodo-4,5-dimethyl-2-phenylthiophene (**2a**) under different conditions *^a^*. 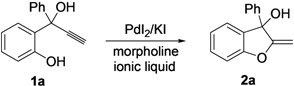

Entry	Ionic Liquid	T (°C)	Conversion of 1a *^b^* (%)	Yield of 2a *^c^* (%)
1	BmimBF_4_	70	80	60
2 *^d^*	BmimBF_4_	70	100	82
3	BmimBF_4_	100	100	86
4 *^e^*	BmimBF_4_	100	100	80
5 *^f^*	BmimBF_4_	100	100	63
6 *^g^*	BmimBF_4_	100	100	65
7	BmimCl	100	100	51
8	BmimPF_6_	100	100	58
9	BmimN(CN)_2_	100	100	63
10	BmimOTf	100	60	23

*^a^* Unless otherwise noted, all cycloisomerization reactions were carried out for 5 h under nitrogen with a substrate concentration of 0.25 mmol of **1a** per mL of ionic liquid. The morpholine:**1a**:KI:PdI_2_ molar ratio was 50:50:5:1. The formation of a complex mixture of products accounted for the difference between substrate conversion and the yield of **2a**. *^b^* Based on isolated unreacted **1a**. *^c^* Isolated yield based on starting **1a**. *^d^* The reaction time was 8 h. *^e^* The reaction was carried out with 1 mol % of PdI_2_. *^f^* The reaction was carried with 4 mol % of KI. *^g^* The reaction was carried with PdCl_2_+5KCl rather than with PdI_2_ + 5KI.

**Table 2:** Recyclable Synthesis of 2-methylene-2,3-dihydrobenzofuran-3-ols (**2**) by PdI_2_/KI-catalyzed cycloisomerization of 2-(1-hydroxyprop-2-ynyl)phenols (**1**) in BmimBF_4_ *^a^*. 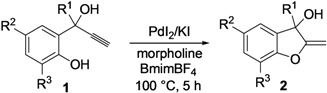

Entry	1	2	Yield of 2 (%) *^b^*						
			Run 1 *^c^*	Run 2 *^c^*	Run 3 *^c^*	Run 4 *^c^*	Run 5 *^c^*	Run 6 *^c^*	Run 7 *^c^*
1	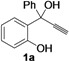	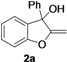	86	85	87	86	85	86	87
2	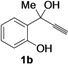	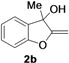	84	86	82	75	72	73	72
3	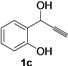	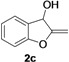	70	71	71	68	70	69	70
4	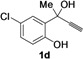	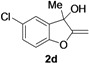	78	80	78	80	81	81	80
5	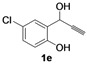	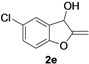	80	81	79	80	78	81	79
6	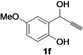	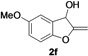	75	77	76	77	75	76	78
7	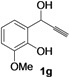	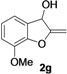	76	76	77	75	78	74	77

*^a^* All reactions were carried out at 100 °C in BmimBF_4_ under nitrogen for 5 h with a substrate concentration of 0.25 mmol of **1** per mL of ionic liquid. The morpholine:**1**:KI:PdI_2_ molar ratio was 50:50:5:1. Conversion of **1** was quantitative in all cases. *^b^* Isolated yield based on starting **1**. *^c^* Run 1 corresponds to the 1st experiment, the next runs to recycles. See text for details.

The method was then extended to other 2-(1-hydroxyprop-2-ynyl)phenols **1b**–**g**, bearing different (either electron-withdrawing or electron-donating) substituents on the aromatic ring and at the benzylic position. As can be seen from the results shown in Table 2, entries 2–7, good yields of the corresponding methylenedihydrobenzofurans **2b**–**g** were consistently obtained. We also checked the catalyst-solvent recyclability in all cases, still with satisfactory results, products being obtained in comparable yields with respect to the parent reactions (Table 2, entries 2–7).

2-Methylene-2,3-dihydrobenzofuran-3-ols **2** are known to be useful precursors for the preparation of functionalized benzofurans [18,42]. In particular, we previously reported that they can easily undergo acid-catalyzed allylic isomerization to give 2-hydroxymethylbenzofurans **3** or acid-catalyzed allylic nucleophilic substitution to give 2-methoxymethylbenzofurans **4** [18]. We have now found that it is possible to directly obtain either benzofurans **3** or **4** in a one-pot fashion by Pd-catalyzed cycloisomerization of **1** in BmimBF_4_, followed by acid catalyzed allylic isomerization or nucleophilic substitution, without the need for isolating compounds **2**. Thus, the reaction mixture resulting from the cycloisomerization process was allowed to cool down to room temperature, and then H_2_SO_4_ in water or in MeOH was added. After 3–18 h at 100 °C, the corresponding benzofuran derivatives **3** and **4** were obtained in high yields (Table 3). Clearly, no IL recyclability was possible for this one-pot transformation.

**Table 3:** Synthesis of 2-hydroxymethylbenzofurans **3** and 2-methoxymethylbenzofurans **4** by one-pot PdI_2_/KI-catalyzed cycloisomerization of 2-(1-hydroxyprop-2-ynyl)phenols ‒ allylic isomerization or allylic nucleophilic substitution *^a^*. 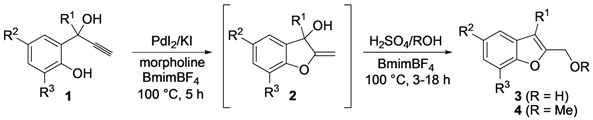

Entry	1	R	t (h)	3 or 4	Yield of 3 or 4 *^b^* (%)
1	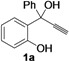	H	3	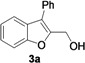	65
2	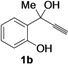	H	3	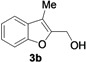	55
3	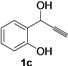	H	3	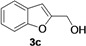	54
4	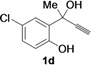	H	3	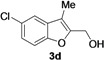	71
5	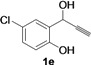	H	3	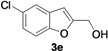	56
6	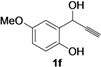	H	3	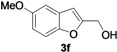	52
7	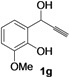	H	3	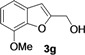	54
8	1a	Me	18	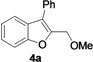	80
9	1b	Me	18	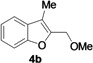	65
10	1c	Me	18	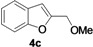	52
11	1d	Me	15	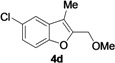	70
12	1e	Me	18	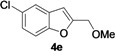	52
13	1f	Me	18	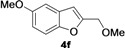	55
14	1g	Me	18	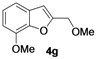	60

*^a^* All cycloisomerization reactions were carried out at 100 °C in BmimBF_4_ under nitrogen for 5 h with a substrate concentration of 0.25 mmol of **1** per mL of ionic liquid. The morpholine:**1**:KI:PdI_2_ molar ratio was 50:50:5:1. The allylic isomerization or allylic nucleophilic substitution was carried out in a one-pot fashion by adding H_2_SO_4_ in water or in MeOH (R = H or Me, respectively; 0.75 M) to the reaction crude deriving from the cycloisomerization reaction, and allowing the resulting mixture to stir a 100 °C for the required time. Conversion of **1** was quantitative in all cases. *^b^* Isolated yield based on starting **1**.

## 3. Experimental

### 3.1. General

Melting points were taken on a Reichert Thermovar apparatus and are uncorrected. ^1^H-NMR and ^13^C-NMR spectra were recorded at 25 °C in CDCl_3_ solutions with a Bruker DPX Avance 300 spectrometer operating at 300 MHz and 75 MHz, respectively, with Me_4_Si as internal standard. Chemical shifts (δ) and coupling constants (*J*) are given in ppm and in Hz, respectively. IR spectra were taken with a JASCO FT-IR 4200 spectrometer. Mass spectra were obtained using a Shimadzu QP-2010 GC-MS apparatus at 70 eV ionization voltage. Microanalyses were carried out with a Carlo Erba Elemental Analyzer Mod. 1106. All reactions were analyzed by TLC on silica gel 60 F_254_ (Merck) or on neutral alumina (Merck) and by GLC using a Shimadzu GC-2010 gas chromatograph and capillary columns with polymethylsilicone + 5% polyphenylsilicone as the stationary phase (HP-5). Column chromatography was performed on silica gel 60 (Merck, 70–230 mesh) or neutral alumina 90 (Merck, 70–230 mesh). Evaporation refers to the removal of solvent under reduced pressure.

### 3.2. Preparation of Substrates

Starting 2-(1-hydroxyprop-2-ynyl)phenols **1** were prepared and characterized as already described [43].

### 3.3. Preparation of Ionic Liquids

Ionic liquid BmimOTf [44] was prepared according to a literature procedure. All other ionic liquids were prepared as previously described [17].

### 3.4. General Procedure for the PdI_2_/KI-Catalyzed Cycloisomerization of 2-(1-Hydroxyprop-2-ynyl)phenols ***1*** to Give 2-Methylene-2,3-Dihydrobenzofuran-3-ols ***2***

To a Schlenk flask containing PdI_2_ (1.8 mg, 5.0 × 10^−3^ mmol), KI (4.2 mg, 2.5 × 10^−2^ mmol) and BmimBF_4_ (1 mL) was added, under nitrogen, a solution of **1** (0.25 mmol) in Et_2_O (1.5 mL). The diethyl ether was eliminated under vacuum, and then morpholine (22.0 mg, 0.25 mmol) was added under nitrogen. The resulting mixture was allowed to stir under nitrogen at 100 °C for 5 h. After cooling, the mixture was extracted with Et_2_O (6 × 2 mL), to separate the product, while the residue, still containing the catalysts dissolved in the IL, was used as such for the recycling experiments (see below). The collected ethereal phases were concentrated, and products **2** were purified by column chromatography on silica gel using hexane-AcOEt from 95:5 to 9:1 as the eluent. The yields obtained in each experiments are reported in Table 2.

*Recycling Procedure.* To the residue obtained as described above, still containing the catalyst dissolved in the ionic liquid, was added under nitrogen a solution of **1** (0.25 mmol) in Et_2_O (1.5 mL). Diethyl ether was removed under vacuum, morpholine (22.0 mg, 0.25 mmol) was added, and then the same procedure described above was followed.

### 3.5. General Procedure for the One-Pot Synthesis of 2-Hydroxymethylbenzofurans ***3*** and 2-Methoxy- methylbenzofurans ***4*** Starting From 2-(1-Hydroxyprop-2-ynyl)phenols ***1***

To a Schlenk flask containing PdI_2_ (1.8 mg, 5.0 × 10^−3^ mmol), KI (4.2 mg, 2.5 × 10^−2^ mmol) and BmimBF_4_ (1 mL) was added, under nitrogen, a solution of **1** (0.25 mmol) in Et_2_O (1.5 mL). The diethyl ether was eliminated under vacuum, and then morpholine (22.0 mg, 0.25 mmol) was added under nitrogen. The resulting mixture was allowed to stir under nitrogen at 100 °C for 5 h. After cooling, a solution of H_2_SO_4_ in ROH (R = H or Me, 0.75 M) (670 μL, 0.5 mmol) was added under nitrogen (together with 100 μL of MeOH when R = Me), and the mixture was allowed to stir at 100 °C for 3 h (R = H) or overnight (18 h, R = Me). After cooling, the mixture was extracted with Et_2_O (6 × 2 mL). The collected ethereal phases were concentrated, and products **3** (R = H) and **4** (R = Me) were purified by column chromatography on silica gel using hexane-AcOEt from 95:5 to 9:1 as the eluent. The yields obtained in each experiment are reported in Table 3.

### 3.6. Characterization of Products

All products **2**, **3**, and **4** were characterized by comparison with the characterization data already reported by us [18].

## 4. Conclusions

In conclusion, we have found that the PdI_2_/KI-catalyzed cycloisomerization of 2-(1-hydroxyprop-2-ynyl)phenols (**1**) to give 2-methylene-2,3-dihydrobenzofuran-3-ols (**2**) can be conveniently carried out in BmimBF_4_ as the reaction medium. When compared with MeOH [18], the use of BmimBF_4_ as the solvent required a higher temperature (100 °C *vs*. 40 °C) and a longer reaction time (5 h *vs*. 2 h); moreover, the yields obtained in BmimBF_4_ were similar or slightly inferior with respect to those obtained under the “classical” conditions (using MeOH as the solvent) [18]. However, the use of the ionic liquid has allowed to recycle the solvent-catalytic system several times, without appreciable loss of catalytic activity. Moreover, in BmimBF_4_, 2-methylene-2,3-dihydrobenzofuran-3-ols (**2**) could be expediently converted into functionalized benzofurans (2-hydroxymethylbenzofurans **3** or 2-methoxymethylbenzofurans **4**) in a one-pot fashion by subsequent acid-catalyzed allylic isomerization or allylic nucleophilic substitution.
